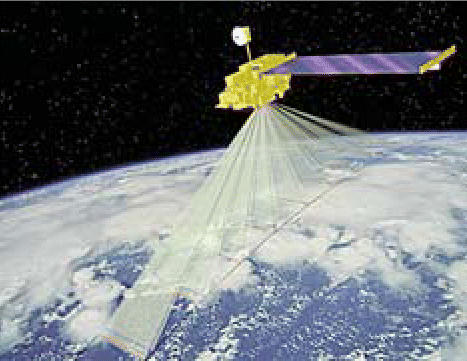# The Beat

**Published:** 2006-02

**Authors:** Erin E. Dooley

## Back-Door Cigarette Marketing?

At a time when marketing restrictions make it harder for tobacco manufacturers to reach the youth market, a number of new candy- and liqueur-flavored tobacco products are hitting the market. A review of internal tobacco industry documents published in the November/December 2005 issue of *Health Affairs* showed that the industry has long sought to target youth through new flavors, with one document stating that young people’s interest in unusual flavors “may indicate new opportunities for enhanced-flavor tobacco products that could leverage [brand’s] current strength among younger adult smokers.” The authors write that flavored cigarettes can promote youth smoking initiation and help young occasional smokers become daily smokers by reducing or masking the unpleasant taste of tobacco smoke. The authors add there is little information on the potential health effects of the flavorings themselves.

## In My Skin

Rates of melanoma, the deadliest skin cancer, continue to climb, more than tripling in Caucasians between 1980 and 2002, according to the American Cancer Society. Now skin cancer experts at the University of Newcastle upon Tyne have developed a novel test that uses a small skin sample and responses to a ten-page questionnaire to produce highly personalized assessments of the risks individuals face from their sun exposure to date. Patients also receive personalized skin protection advice and can re-take the test to see how changes they’ve made have affected their skin cancer risk. The “skinphysical” test was launched at British clinics in the autumn of 2005.

## The Healing Quiet

A new study from The Johns Hopkins University shows that a noisy hospital environment may make patients sicker and lead to higher stress levels and burnout among staff. The study, presented at the 2005 annual meeting of the Acoustical Society of America, found that hospital noise levels worldwide have grown steadily over the past five decades and now on average exceed WHO hospital noise guidelines. This disturbs those within the hospital’s confines, raises the risk of medical errors, and can even slow the pace of healing and contribute to lapses in short-term memory. Two possible solutions are to equip hospital personnel with hands-free personal communicators (eliminating the need for loudspeakers) and to wrap fiberglass insulation with an antibacterial fabric to form a sound-absorbent tile for ceilings and walls.

## New Guidelines for Pediatric Asthma

More than 6 million U.S. children have asthma, the leading cause of school absenteeism attributable to chronic conditions and the third leading cause of hospitalization among children under age 15. In November 2005, the National Environmental Education & Training Foundation released *Environmental Management of Pediatric Asthma: Guidelines for Healthcare Providers*. Funded by the NIEHS, the peer-reviewed guidance was built on current best practices and includes competencies for managing environmental asthma triggers in pediatric care, an environmental history form for clinicians to use, and intervention guidelines and fliers for specific triggers such as dust mites, cockroaches, and mold spores. Incorporating these guidelines into medical and nursing curricula could give future generations of primary care providers the tools to better manage pediatric asthma.

## Own Private Kyoto

Despite producing 24% of all greenhouse gas emissions worldwide, the United States has not signed on to the Kyoto Protocol to reduce greenhouse gases. An analysis in the 17 November 2005 *Nature* shows, however, that as much as one-third of the U.S. population lives in areas that have adopted their own climate change abatement policies. Together, these regions contribute almost half of the U.S. GDP, a slightly larger share of the global GDP than Japan, the world’s second largest economy. The authors warn that compliance could be challenging, though, especially since there are currently no mechanisms for enforcement.

## Software for Sorting Satellite Images

NASA satellites generate enough data daily to fill 1,500 copies of the *Encyclopedia Britannica*, but satellite data maps often have blank spots where a satellite wasn’t able to record data on a particular day. Now statisticians at The Ohio State University have developed new software that can help researchers rapidly process incoming data to produce complete, detailed maps. In an example given by lead statistician Noel Cressie, it could take one person 500 years to fill in the gaps in a map depicting the thickness of the ozone layer, while the same job would take three minutes with the new software. The software also calculates a measure of map precision.

## Figures and Tables

**Figure:**
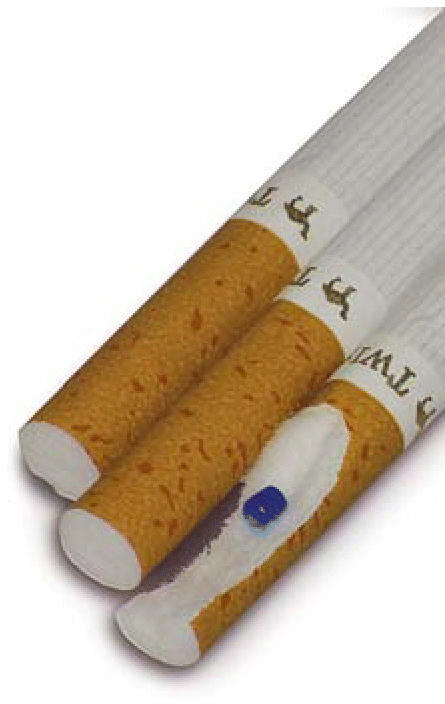


**Figure:**
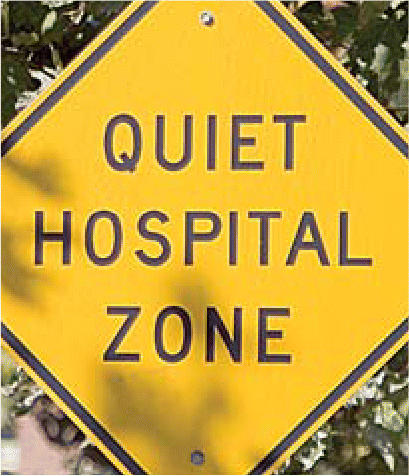


**Figure:**
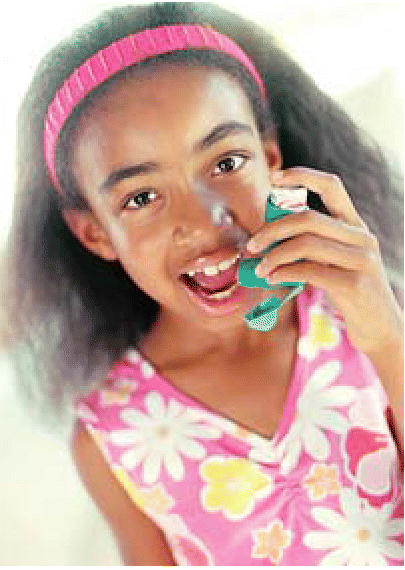


**Figure:**